# When Acute Epstein-Barr Virus Mimics HIV: A Case of False-Positive p24 Antigen and Low-Level HIV Antibody Reactivity

**DOI:** 10.7759/cureus.92395

**Published:** 2025-09-15

**Authors:** Smriti Chaudhary, Pranav Jha

**Affiliations:** 1 Acute Medicine, Imperial College Healthcare NHS Trust, London, GBR; 2 Gastroenterology, Imperial College Healthcare NHS Trust, London, GBR

**Keywords:** acute ebv infection, acute hepatitis, diagnostic pitfall, false positive hiv, hiv screening, p24 antigen, viral cross-reactivity

## Abstract

False-positive HIV screening results are uncommon but clinically significant, often leading to patient anxiety and diagnostic uncertainty. Acute Epstein-Barr virus (EBV) infection is a recognised but rare cause of cross-reactivity with fourth-generation HIV assays, particularly p24 antigen and low-level HIV antibody reactivity.

We report a 57-year-old male with a history of hypothyroidism and atrial fibrillation who presented with sore throat, tender cervical and occipital lymphadenopathy, diffuse rash, dark urine, and fatigue. Initial investigations showed lymphocytosis and marked transaminitis. Fourth-generation HIV testing was repeatedly reactive, with positive p24 antigen and low-level HIV-1/2 antibody reactivity across three separate samples. In contrast, HIV-1 RNA and HIV-2 RNA viral loads were consistently undetectable (<20 copies/mL). EBV serology demonstrated viral capsid antigen (VCA) IgM positivity with transient EBV DNA detection, and the clinical presentation was consistent with acute EBV infection. Additional viral serologies, including cytomegalovirus (CMV), varicella-zoster virus (VZV), measles, parvovirus, and syphilis, were negative. Monkeypox PCR was weakly positive once but negative on repeat testing. The patient’s wife, who was pregnant, tested negative for HIV. He improved with supportive care, and follow-up demonstrated normalisation of liver function tests and persistently negative HIV serology, confirming a false-positive HIV result in the setting of acute EBV infection.

This case is significant because it illustrates a rare but important diagnostic pitfall: acute EBV infection mimicking acute HIV. The implications of a false HIV diagnosis are profound, carrying psychological, social, and clinical consequences, heightened in this case by the patient’s pregnant wife. The discrepancy between reactive screening assays and undetectable HIV-1 and HIV-2 RNA underscores the importance of confirmatory testing and clinical correlation.

Clinicians should be aware that acute EBV infection may result in false-positive HIV p24 antigen and antibody reactivity. Reporting such cases adds to the limited literature and serves as an important reminder to interpret discordant HIV results with caution to avoid misdiagnosis and unnecessary psychological burden.

## Introduction

Accurate and timely diagnosis of HIV infection is essential for initiating treatment and preventing onward transmission. Modern diagnostic algorithms begin with a fourth-generation HIV-1/2 antigen-antibody (Ag/Ab) combination immunoassay, followed by an HIV-1/2 differentiation assay and, if needed, nucleic acid testing (NAT) when results are discordant [1,2]. Fourth-generation assays have significantly reduced the diagnostic window period by detecting both HIV antibodies and p24 antigen, enabling detection as early as two weeks post-exposure [3,4]. Specifically, fourth-generation HIV antigen-antibody combination assays reduce the window period by detecting HIV-1 p24 antigen as early as 14-16 days post-infection and HIV antibodies at approximately 21 days post-infection.

Despite their high sensitivity, false-positive results remain possible, cited in the literature between 0.08% and 0.16% in large cohorts. Causes include autoimmune disorders; recent vaccination, such as influenza and tetanus/diphtheria/pertussis (Tdap) boosters; and acute viral infections, during which polyclonal B-cell activation and heterophile antibody production can result in non-specific immunoassay binding [5,6]. Epstein-Barr virus (EBV) infection is particularly noteworthy: by triggering robust B-cell activation and producing heterophile antibodies, EBV can cause cross-reactivity on sensitive HIV screening assays, sometimes yielding false-positive or inconclusive results [7]. Reports of false-positive HIV results in the context of acute EBV infection remain uncommon, with only a limited number of cases described in the literature. Given the clinical overlap between acute EBV and primary HIV infection, such as fever, lymphadenopathy, rash, and transaminitis, misinterpretation of reactive screening assays can occur. Documenting additional cases is therefore important to raise awareness of this diagnostic pitfall and to reinforce the need for confirmatory NAT before establishing a diagnosis of HIV.

## Case presentation

A 57-year-old man with a history of hypothyroidism, attention-deficit/hyperactivity disorder, and paroxysmal atrial fibrillation (on apixaban) presented on July 2, 2025 with five to seven days of sore throat, tender cervical and occipital lymphadenopathy, diffuse pruritic rash, fatigue, dark urine, nausea/vomiting, and dysuria. He denied high-risk behaviours for HIV acquisition. Of note, his spouse was pregnant, heightening the psychosocial impact of preliminary results.

On examination, he was haemodynamically stable. There was bilateral tonsillar inflammation with a left tonsillar ulcer, tender submandibular and posterior auricular nodes, and a widespread maculopapular rash with scattered vesicular lesions across the trunk and upper limbs. No meningism or focal abdominal tenderness was present.

In accordance with the hospital’s standard policy and the NHS opt-out blood-borne virus testing programme, the patient underwent routine HIV testing. A fourth-generation HIV Ag/Ab screen was reactive on two separate blood samples collected 48 hours apart. The sample taken on Day 1 reported p24 antigen reactivity with low-level HIV-1/2 antibody signals (Table 1). The test was repeated on Day 3 of admission, when HIV-1 RNA and HIV-2 RNA by NAT remained undetectable (Table 2) despite detectable HIV antibodies and p24 antigen, arguing strongly against acute HIV infection. Further, HIV-1 RNA was not detected across four independent gene targets (gag, pol, LTR, env), covering both group M and O subtypes (Table 3). These findings strongly excluded acute HIV infection despite the repeated serologic reactivity [8].

**Table 1:** HIV results showing positive p24 antigen detection and borderline antibody levels on the day of admission (Day 1). Ag: Antigen; Ab: Antibody; NAT: Nucleic acid test; RLU: Relative light units. Note: Non-reactive defined as RLU/CO < 1.0.

Parameter	Patient result	Reference value	Units
HIV 1/2 Ag/Ab (Screen, RLU)	1.38	Non-reactive < 1.0	RLU
Repeat HIV Screen (RLU)	1.24	Non-reactive < 1.0	RLU
HIV Confirmation Ab	8.4	Non-reactive < 1.0	RLU
HIV Confirmation p24 Ag	34.53	Non-reactive < 1.0	RLU
HIV-2 Confirmation Ab	3.49	Non-reactive < 1.0	RLU

**Table 2:** Repeat testing on Day 3 of admission shows persistently detectable HIV antibodies and p24 antigen, with negative HIV-1 and HIV-2 RNA levels. Ag: Antigen; Ab: Antibody; NAT: Nucleic acid test; RLU: Relative light units. Note: Non-reactive defined as RLU/CO < 1.0.

Parameter	Patient result	Reference value	Units
HIV-1 RNA Viral Load (NAT, plasma)	Not detected (<20)	Not detected (<20)	copies/mL
HIV-2 RNA (NAT, plasma)	Not detected	Not detected	copies/mL
HIV 1/2 Ag/Ab (Screen, RLU)	1.21	Non-reactive < 1.0	RLU
HIV Confirmation Ab	7.9	Non-reactive < 1.0	RLU
HIV Confirmation p24 Ag	32.02	Non-reactive < 1.0	RLU
HIV-2 Confirmation Ab	3.2	Non-reactive < 1.0	RLU

**Table 3:** Negative HIV-1 RNA results across four independent gene targets (gag, pol, LTR, env). NAT: Nucleic acid test; gag/pol/LTR/env: HIV-1 gene targets tested; copies/mL: Copies per milliliter.

Gene target	Patient result	Reference value	Units
HIV-1 gag DNA/RNA	Not detected	Not detected	copies/mL
HIV-1 pol DNA/RNA	Not detected	Not detected	copies/mL
HIV-1 LTR DNA/RNA	Not detected	Not detected	copies/mL
HIV-1 env DNA/RNA	Not detected	Not detected	copies/mL

Given the symptoms of sore throat and lymphadenopathy, the patient was also tested for Epstein-Barr virus (EBV) infection, and interestingly EBV testing demonstrated VCA IgM positivity with transient EBV DNA detection, aligning with acute EBV infection as the unifying diagnosis (Table 4).

**Table 4:** EBV testing shows positive VCA IgM with detectable EBV DNA, supporting active EBV infection. EBV: Epstein-Barr virus; VCA: Viral capsid antigen; IgM: Immunoglobulin M; IU/mL: International units per milliliter.

Parameter	Patient result	Reference value	Units
EBV VCA IgM (serology)	Detected	Not detected	-
EBV DNA PCR (plasma)	71	Not detected	IU/mL

Because the rash included vesicular lesions, the patient also underwent mpox (monkeypox) PCR testing. One early swab returned a weak positive (high cycle threshold/late Ct). In line with public health guidance, that late-Ct positives with low pre-test probability warrant re-extraction and repeat testing to exclude false positivity, additional samples from lesions were collected and tested; all repeat mpox PCRs were negative, and mpox precautions were discontinued [9-11].

He received supportive care only. By July 11, 2025, as his repeat mpox test result came back negative, his symptoms were improving, and the rash was fading, he was discharged.

At post-discharge review, pruritus and lymphadenopathy had continued to improve; repeat HIV Ag/Ab testing was non-reactive and HIV-1/2 RNA remained undetectable, corroborating a false-positive HIV screen in the setting of acute EBV. As it was noted during admission that the alanine aminotransferase (ALT) was raised (Table 5), an abdominal ultrasound performed at discharge also showed a normal liver with mild fatty infiltration and no biliary obstruction.

**Table 5:** Rise and subsequent normalisation of ALT, consistent with acute EBV hepatitis. ALT: Alanine aminotransferase; U/L: Units per liter. Reference range: < 40 U/L (normal).

Time point	ALT result	Reference value	Units
Day 1	293	< 40	U/L
Peak	440	< 40	U/L
Recovery	360	< 40	U/L
Follow-up	43	< 40	U/L

Concurrent biochemistry demonstrated gradual improvement of his markers, and Table 5 shows normalisation of ALT, suggestive of resolution of acute hepatitis, further supporting a diagnosis of acute EBV hepatitis.

His spouse tested HIV-negative and later delivered a healthy infant without complications. The overall clinical and laboratory course supported acute EBV infection with cross-reactivity on HIV screening; the transient weak mpox signal was deemed a false positive after repeat testing [8-13].

## Discussion

HIV diagnostic challenges in the modern era

HIV diagnostic strategies have evolved considerably over the past three decades. First-generation assays detected only IgG antibodies, leaving a prolonged window period of up to 12 weeks. By contrast, fourth-generation assays detect both HIV-1/2 antibodies and the p24 antigen, narrowing the window period to approximately two weeks [1,2]. Fifth-generation assays go further by separately reporting reactivity to HIV-1 antibodies, HIV-2 antibodies, and HIV-1 p24 antigen [3]. While these developments enhance early detection, they also increase assay complexity and the possibility of false positives, particularly in populations with low HIV prevalence. The range of false-positive rates for fourth- and fifth-generation HIV antigen-antibody (Ag/Ab) assays in clinical studies is typically 0.07% to 0.28% in general screening populations, with specificity consistently above 99.8%. In low-prevalence settings, the proportion of reactive screens that are ultimately false positive can be substantially higher; for example, one study found that 12.5% of positive screens were false positives after confirmatory testing [4].

In the case presented here, three separate samples collected over 48 hours demonstrated low-level reactivity for both HIV-1/2 antibodies and p24 antigen. On initial review, these findings could easily be interpreted as acute HIV seroconversion. However, the absence of HIV RNA across four independent gene targets (gag, pol, LTR, env), together with a negative HIV-2 RNA result, definitively excluded true infection. These results highlight the critical role of NAT as the “tie-breaker” when serology is discordant with clinical features [5,6].

EBV infection as a source of HIV false positives

EBV is a ubiquitous herpesvirus associated with infectious mononucleosis, lymphoproliferative disorders, and a variety of malignancies. A hallmark of acute EBV infection is polyclonal B-cell activation and heterophile antibody production [14]. This immune activation increases the likelihood of cross-reactivity in serological assays.

Several mechanisms have been proposed to explain false-positive HIV serology during acute EBV infection. The presence of cross-reactive epitopes has been suggested, as acute EBV infection can induce polyclonal B-cell activation and heterophile antibodies, leading to non-specific cross-reactivity with HIV antigens; false-positive immunoblot reactivity in this setting often involves gp41 and p24 bands [15]. A second proposed mechanism is generalized polyclonal B-cell activation, whereby EBV drives massive, non-specific immunoglobulin production that increases the likelihood of assay interference [16]. Finally, high levels of circulating antigen-antibody immune complexes during acute EBV infection may non-specifically trigger chemiluminescent or enzyme-based detection systems, resulting in spurious HIV reactivity [17].

In our patient, the constellation of EBV VCA IgM positivity, atypical lymphocytosis, transient EBV DNA detection, and transaminitis was highly consistent with primary EBV infection. The timeline of symptoms (sore throat, lymphadenopathy, rash, fatigue) also fit the classic syndrome [16]. Thus, EBV represents the most plausible cause of the discordant HIV results.

Diagnostic and psychosocial implications

False-positive HIV screening can have profound consequences. Patients may experience significant psychological distress, especially if preliminary results are disclosed before confirmatory testing. In this case, the stakes were higher because the patient’s spouse was pregnant, raising urgent questions around maternal and neonatal HIV transmission risk. Such scenarios underscore the need for careful communication: preliminary results should be presented as “reactive” rather than “positive,” with clear emphasis on the need for confirmatory testing [18].

False positives also carry public health implications. Inappropriate partner notification, unnecessary antiretroviral initiation, and unwarranted caesarean delivery in pregnant women are documented risks when discordant HIV results are misinterpreted [18]. Furthermore, the financial and resource burden of repeated confirmatory testing is non-trivial in health systems where HIV prevalence is low and the positive predictive value of screening is modest [19].

Mpox PCR false positivity: a second confounder

This case is made more complex by an initial weakly positive mpox PCR with a late cycle threshold (Ct). In the context of the 2022 global mpox outbreak, late-Ct positives in low-prevalence settings have been increasingly recognised as false positives [20]. Guidance from both CDC and United Kingdom Health Security Agency (UKHSA) stresses repeat testing in such cases to avoid overdiagnosis [20]. Our patient’s subsequent negative mpox PCRs confirmed the initial result was spurious. In the case of our patient, this initial false-positive result necessitated a prolonged inpatient admission while further testing was awaited, leading to increased healthcare costs and psychological anxiety.

The co-occurrence of two independent false-positive viral tests (HIV serology and mpox PCR) in the same patient is exceptionally rare in the literature. To our knowledge, no case report or study documents the co-occurrence of both a false-positive HIV serology and a false-positive monkeypox PCR result in the same patient. This dual diagnostic pitfall reinforces the principle that laboratory findings must be interpreted in the clinical and epidemiological context.

Why this case is noteworthy

To our knowledge, only a few published cases have documented this unique constellation of findings. Our patient demonstrated simultaneous low-level HIV antibody and p24 antigen reactivity that persisted across multiple samples. This was definitively excluded by multi-target HIV NAT and HIV-2 RNA testing. The phenomenon occurred in the setting of acute EBV infection and was further complicated by an additional false-positive mpox PCR, which contributed to diagnostic confusion.

This case therefore contributes uniquely to the literature by illustrating how multiple layers of false positivity can mislead clinicians. It emphasises the necessity of confirmatory NAT, the importance of considering EBV as a benign cause of HIV false positivity, and the risks of over-reliance on single assay results in low-prevalence populations.

## Conclusions

This case highlights the diagnostic pitfalls of modern serological testing in the context of acute viral infection. Our patient exhibited repeatedly reactive fourth-generation HIV screening assays, including p24 antigen reactivity, yet was conclusively HIV-negative on multi-target HIV-1 RNA and HIV-2 RNA testing. The ultimate diagnosis of acute EBV infection explained the discordant findings through cross-reactivity and polyclonal B-cell activation, mechanisms well-documented but often overlooked in current clinical practice.

Further, this case contributes valuable insight to a limited body of evidence. Our patient demonstrated repeated reactivity to both p24 antigen and low-level HIV-1/2 antibodies on fourth-generation screening, yet confirmatory testing revealed persistently undetectable HIV-1 and HIV-2 RNA. When interpreted within established algorithmic frameworks, this scenario aligns with false positivity due to assay cross-reactivity in the setting of acute EBV infection rather than true HIV infection. The stakes were high: a potential HIV diagnosis would have carried significant psychosocial implications, notably because the patient’s spouse was pregnant. The case underlines the critical importance of confirmatory testing and nuanced clinical interpretation.

The case is further distinguished by an initial false-positive monkeypox PCR, which was later negated on repeat testing. The coexistence of two separate false-positive viral diagnostic results in a single patient underscores the necessity of careful clinical correlation, confirmatory molecular testing, and cautious interpretation of preliminary results. For clinicians, this reinforces the importance of adhering to diagnostic algorithms, avoiding premature disclosure of provisional results, and considering alternative infectious explanations such as EBV when encountering unexpected HIV serological reactivity.

## References

[1] (2025). Centers for Disease Control and Prevention: Laboratory testing for the diagnosis of HIV infection: updated recommendations. https://www.cdc.gov/mmwr/preview/mmwrhtml/rr6303a1.htm.

[2] (2025). Association of Public Health Laboratories (APHL): Suggested reporting language for the HIV laboratory diagnostic testing algorithm. https://www.aphl.org/aboutAPHL/publications/Documents/ID-2019Jan-HIV-Lab-Test-Suggested-Reporting-Language.pdf.

[3] Pandori MW, Hackett J Jr, Louie B, Vallari A, Dowling T, Liska S, Klausner JD (2009). Assessment of the ability of a fourth-generation immunoassay for human immunodeficiency virus (HIV) antibody and p24 antigen to detect both acute and recent HIV infections in a high-risk setting. J Clin Microbiol.

[4] Alexander TS (2016). Human immunodeficiency virus diagnostic testing: 30 years of evolution. Clin Vaccine Immunol.

[5] Lavoie S, Caswell D, Gill MJ (2018). Heterophilic interference in specimens yielding false-reactive results on the Abbott 4th generation ARCHITECT HIV Ag/Ab Combo assay. J Clin Virol.

[6] Ly TD, Laperche S, Brennan C (2004). Evaluation of the sensitivity and specificity of six HIV combined p24 antigen and antibody assays. J Virol Methods.

[7] Mbopi-Keou FX, Ndjoyi-Mbiguino A, Talla F (2014). Association of inconclusive sera for human immunodeficiency virus infection with malaria and Epstein-Barr virus infection in Central Africa. J Clin Microbiol.

[8] Rosenberg NE, Pilcher CD, Busch MP, Cohen MS (2015). How can we better identify early HIV infections?. Curr Opin HIV AIDS.

[9] (2025). Centers for Disease Control and Prevention: Guidelines for collecting and handling specimens for mpox testing. https://www.cdc.gov/mpox/hcp/diagnosis-testing/collecting-specimens.html.

[10] (2025). UK Health Security Agency (UKHSA): Mpox: diagnostic testing. (2025). GOV.UK. https://www.gov.uk/guidance/monkeypox-diagnostic-testing.

[11] Li Y, Olson VA, Laue T, Laker MT, Damon IK (2006). Detection of monkeypox virus with real-time PCR assays. J Clin Virol.

[12] Delaney KP, Hanson DL, Masciotra S, Ethridge SF, Wesolowski L, Owen SM (2017). Time until emergence of HIV test reactivity following infection with HIV-1: implications for interpreting test results and retesting after exposure. Clin Infect Dis.

[13] Fiebig EW, Wright DJ, Rawal BD (2003). Dynamics of HIV viremia and antibody seroconversion in plasma donors: implications for diagnosis and staging of primary HIV infection. AIDS.

[14] Thorley-Lawson DA, Gross A (2004). Persistence of the Epstein-Barr virus and the origins of associated lymphomas. N Engl J Med.

[15] Sayre KR, Dodd RY, Tegtmeier G, Layug L, Alexander SS, Busch MP (1996). False-positive human immunodeficiency virus type 1 western blot tests in noninfected blood donors. Transfusion.

[16] Dunmire SK, Hogquist KA, Balfour HH (2015). Infectious mononucleosis. Curr Top Microbiol Immunol.

[17] Tortosa-Carreres J, Lloret-Sos C, Sahuquillo-Arce JM (2024). Evaluating the diagnostic performance of Liaison® chemiluminescence assay as screening tool for detection of acute Epstein-Barr infection: a comparative study. Diagn Microbiol Infect Dis.

[18] Bhattacharya R, Barton S, Catalan J (2008). When good news is bad news: psychological impact of false positive diagnosis of HIV. AIDS Care.

[19] Bentsen C, McLaughlin L, Mitchell E (2011). Performance evaluation of the Bio-Rad Laboratories GS HIV Combo Ag/Ab EIA, a 4th generation HIV assay for the simultaneous detection of HIV p24 antigen and antibodies to HIV-1 (groups M and O) and HIV-2 in human serum or plasma. J Clin Virol.

[20] Minhaj FS, Petras JK, Brown JA (2022). Orthopoxvirus testing challenges for persons in populations at low risk or without known epidemiologic link to monkeypox - United States, 2022. MMWR Morb Mortal Wkly Rep.

